# Effects of fluoride versus amorphous calcium phosphate solutions on enamel microhardness of white spot lesions: An in-vitro study

**DOI:** 10.4317/jced.54448

**Published:** 2019-03-01

**Authors:** Fahimeh Farzanegan, Seyed Morteza-Saadat-Mostafavi, Hamideh Ameri, Hossein Khaki

**Affiliations:** 1 Associate professor, Oral & Maxillofacial Diseases Research Center, Department of Orthodontics, School of Dentistry, Mashhad University of Medical Sciences, Iran,, Mashhad; 2 Assistant Professor, Faculty of Dentistry, Department of Orthodontics, Kashan University of Medical Sciences, , Iran, Kashan; 3 Specialists in operative dentistry, private practice; 4 Resident of orthodontics, Student Research Committee, Department of Orthodontics, Faculty of Dentistry, Mashhad University of Medical Sciences, Mashhad, Iran

## Abstract

**Background:**

Development of white spot lesions around orthodontic fixed orthodontic appliances is a common finding, especially in patients with poor oral hygiene. One of the conservative interventions for regression of these lesions is using chemical solutions. The current study aimed to compare the effectiveness of fluoride and amorphous calcium phosphate (ACP) on microhardness improvement of affected enamel.

**Material and Methods:**

Forty-five intact human incisor teeth were selected and randomly divided into 3 groups of 15. Fluoride group, ACP group and artificial saliva group (control group). Inducing of white spot lesion was done by PH-cycling model. Samples of the first and second group were submerged into 0.05% fluoride and 0.05% ACP solutions respectively for one minute a day. The rest of the time, all specimens were put in artificial saliva, which was incubated in 37 °c temperature. Microhardness of specimens was assessed by Vickers microhardness test in three stages: 1: Baseline microhardness assessment that was done before induction of white spot lesion, 2: Secondary microhardness assessment that was done after induction, 3: Final microhardness assessment that was done after chemical treatment. The SPSS 11.5 software was used for statistical analysis and *p*< 0.05 was considered as significant.

**Results:**

Microhardness of specimens in the fluoride and ACP groups had significantly improved after the treatment (between secondary assessment and final assessment). In the control group, no significant improvements were observed. In final assessment, there were significant differences between the ACP and control groups, but no significant differences were found neither between the fluoride and ACP, nor the Fluoride and control groups.

**Conclusions:**

According to the current study, both 0.05% ACP and 0.05% fluoride solutions enhanced enamel micro-hardness in treatment of white spot lesion.

** Key words:**Microhardness, amorphous calcium phosphate, fluoride, white spot lesion.

## Introduction

White spot lesions or enamel decalcification is the clinical manifestation of early enamel caries and attributes to the prolonged retention and accumulation of dental bacterial plaque. The white appearance of these lesions is the result of different optical reflections due to mineral loss in the surface or subsurface enamel (1). A great number of orthodontic patients may face these lesions (2) and following the removal of orthodontic appliances, white spots may be observed in the following sites: surface adjacent to the brackets which is covered by resin, at the junction of etched enamel surface and bonding resin (3), cervical margin of the teeth, and beneath the bands (particularly where the cementing medium has washed out) (4). There is consensus that white spot development seems to be related to three main factors: 1) plaque retention, especially on the gingival side of orthodontic appliances, 2) lack of appropriate oral hygiene by the patient, 3) inherent resistance of each individual (5). Although there is linear correlation between plaque accumulation and development of white spot lesions around orthodontic appliances (6), but there are other factors which aggravate this process. Investigations revealed that after use of bonded orthodontic appliances, an increase in streptococci, veillonella, lactobacilli, staphylococci, and yeast was observed (especially oral lactobacilli and Streptococcus mutans which are well-known for their cariogenic characteristics) (7). Evaluations using scanning electron microscopy (SEM) indicated that such bacterial plaque around orthodontic bands, could lead to marked etching of underneath enamel (8). Despite this evidence, there are studies showing that no correlation exists between orthodontic treatment and increased incidence of dental caries (9). It is important for white spot lesions to be prevented, but fortunately these demineralized areas can be remineralized easily, because the outermost layer of enamel remains intact and the underneath layer can be remineralized by absorbing minerals (10). Fluoride is a protective agent which can prevent or reverse dental caries via the following mechanisms: 1) adsorption to minerals of the enamel and enhancing protective mechanisms against acid dissolution 2) counteracting bacterial enzymes, which leads to bacterial plaque inactivity, 3) speeding up the remineralization process by attracting calcium ions in the partially demineralized subsurface crystals in carious lesions (11). Investigations on the effects of fluoride rinsing on reducing white spots in the orthodontic population revealed that, significant dose response relationship exists between rinsing with fluoride solution and enamel white spot reduction (5,12). New method of fluoride application (e.g. fluoride varnish) has also shown to be effective in reversing white spot lesions after debonding (13). Amorphous calcium phosphate (ACP) is an intermediate product during formation of mineralized tissue. Its features are the same as crystalized hydroxyapatite, but it is smaller and can grow in aqueous media and transform into apatite and octacalcium phosphate (14). ACP buffers the free calcium and phosphate ion activities which help maintain the supersaturation states, resulting in enhancement of remineralization and suppression of demineralization (15). Also, assessments demonstrated that Casein phosphopeptide-amorphous calcium phosphate (CPP-ACP) can significantly increase the level of calcium and phosphate in supragingival plaque dose-dependently (16). Few studies indicated that post-orthodontic white lesions are treated successfully with ACP (17,18), although there are investigations that mention no advantage for use of ACP in treatment of post-orthodontic white spot lesions (19).

The aim of the current study was to compare the effectiveness of 0.05% fluoride solution and 0.05% ACP solution on treatment of white spot lesion in an accurate lab trial.

## Material and Methods

-Sample size calculation 

**Figure 1:**
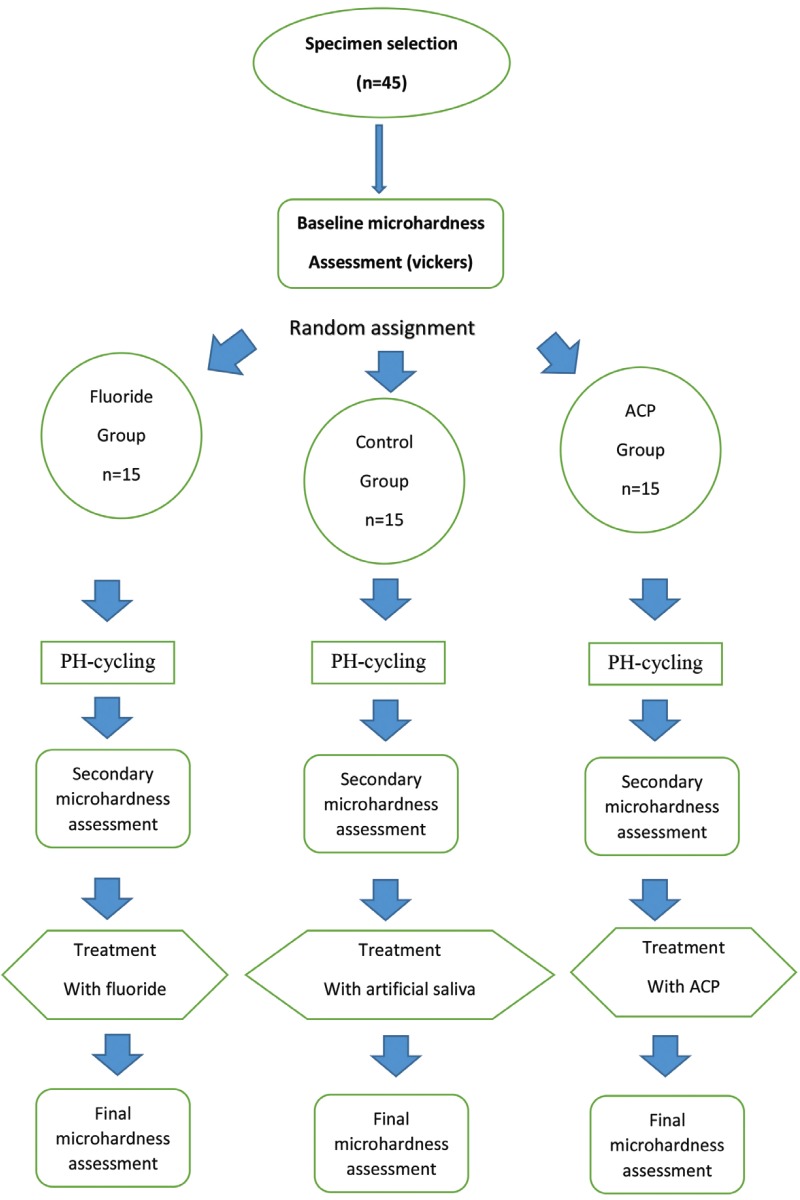
Schematic flowchart of the study protocol.

Data from study of Panich *et al.* (20) provided estimates of standard deviation of percentage changes in enamel hardness following treatment with CPP-ACP paste and artificial saliva. Mean hardness changes after immersion in CPP-ACP and artificial saliva were 326.6 (SD, 11.57) and 296.7 (SD, 21.38) respectively. To achieve a study at an alpha level of 0.01 and beta level of 0.1, the required number of subjects was calculated to be 10 in each group. In order to improve the accuracy of the study we put 15 specimen in each group. In figure 1 study flowchart has shown schematically.

-Specimen preparation 

Forty-five intact human central incisors without caries, defects or restoration were selected and after debridement, placed in Thymol antiseptic solution for 24 hours and then stored in normal saline.

-Microhardness assessment

Enamel surface was polished with sandpaper with grit size of 300, 600, and 1000 consecutively to remove the outer 50 µm (21) microhardness assessment was done using the MATSUZAWA microharness test machine (MHT2, Japan) with a knoop diamond under load of 300 g for five second. Three notches were made longitudinally on the polished surface of the enamel. After polishing the enamel surfaces, Baseline microhardness assessment of each specimen was done to help calculating the microhardness changes following demineralization.

-Developing of White spot lesions 

Specimens were randomly divided into three groups of 15. Proximal, lingual and root surfaces of all teeth were covered by acid-resistant varnish and the apical foramen was covered with dental sticky wax. In order to demineralize the enamel surface of subjects, the PH-cycling model was performed. This model was described for the first time by Featherstone *et al.* (21) to establish a set of conditions that preserve the enamel surface and produce primary carious lesions for lab trials. In the current study, all specimens were submitted to the pH-cycling model at 37˚C, for five days. The procedure consisted of six hours of soaking in demineralizing solution (2.0 mmol/l NaH2PO4.H2O and 2.0 mmol/l NaH2PO4.H2O in 75 mmol/l acetate buffer, pH 4.7; 0.04 mg F/ml as NaF, 2.2 ml/mm2) and 18 hours in the remineralizing solution (0.9 mmol/l NaH2PO4.H2O; 1.5 mmol/l Ca (NO3)2.H2O; 150 mmol/l KCl in 0.02 mol/l cacodylic buffer, pH 7.0; 0.05 mg F/ml as NaF, 1.1 ml/mm2). After that, subjects remained in remineralization solution for another two days and were then stored in artificial saliva for the following secondary microhardness assessment.

-White spot treatment

Three different Medias were prepared for white spot treatment. The fluoride rinse (a neutral 0.05% sodium fluoride, Oral B, California, USA), ACP solution (0.05% Ca3 (PO4)2 in 2% HPMC as a buffer) and modified Fusayama artificial saliva solution ( KCl (400 mg/l), NaCl (400 mg/l), NaH2PO4•H2O (690 mg/l), CaCl2•2H2O (795 mg/l), Na2S•9H2O (5 mg/l), KSCN (300 mg/l), and urea (1000 mg/l). Samples of the first and second groups were placed in 10 cc of fluoride rinse and ACP solution respectively for one minute a day on the vibrating machine (to resemble the mouth washing action). At other times, these specimens in addition to the third group specimens were kept in artificial saliva, which was incubated in 37˚C. After 10 weeks of performing this procedure for the groups, they underwent the final microhardness assessment.

-Statistical analysis 

After evaluating the normality of data with the Kolmogorov–Smirnov test, multiple measurement tests were done to indicate any significant differences between baselines, secondary and final microhardness assessments in each group. In order to determine the differences of microhardness assessments between groups, the analysis of variance test (ANOVA) was used. Statistical Analysis was performed using SPSS 11.5 (SPSS, Inc, Chicago, IL), and the P values less than 0.05 were considered as significant.

## Results

 Table 1 demonstrates the results of microhardness assessment in the three groups at three different times. In order to compare the changes between groups, the analysis of the variance test (ANOVA test) was performed and it showed that in the final microhardness assessment, there were significant differences between the groups. Then the Tukey test was run to determine which groups were different in microhardness alterations following the treatment. Data of this test demonstrated that there was no significant difference in microhardness between the ACP and fluoride groups. Also, no significant difference was found between the fluoride and control groups, but the ACP group showed significant improvement in final microhardness assessment, compared to the control group. Intragroup analysis (among baseline, secondary and final microhardness assessment in each group) with repeated measurement test revealed that there were significant differences between baseline, secondary and final microhardness assessments in fluoride group and ACP group (*p* value = 0.049 and *p* value = 0.017 respectively). Further analysis showed that in fluoride group significant differences existed between secondary and final assessment (*p* value = 0.023), while in ACP group significant differences observed between baseline and final assessments and also secondary and final assessments (*p* value = 0.016 and *p* value = 0.001 respectively). No significant difference was seen among baseline, secondary and final microhardness assessment of control group.

**Table 1:**
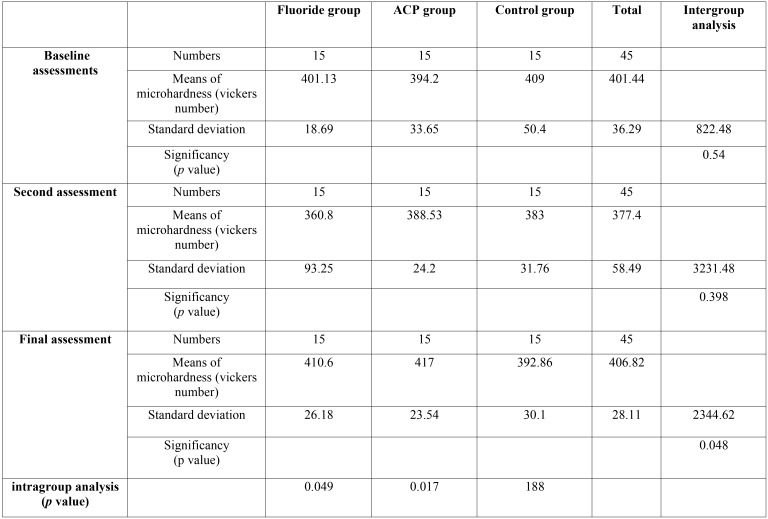
Descriptive statics of each group, Results of Analysis of variance between groups to compare differences among Fluoride, ACP and control groups (Intergroup analysis), Results of multiple measurement test in each group to compare differences among baseline, secondary and final microhardness assessments (Intragroup analysis).

## Discussion

White spot lesions are common problems in many orthodontic patients with fixed appliances, and different preventive measures have been suggested to reduce their incidence (22). Local fluoride exposure showed to be effective in prevention of such enamel defects (17). However, less is known about the treatment and management of occurred lesions (23). The current study was designed as a superiority test to determine whether or not calcium phosphate has more advantages in treatment of white spot lesions than fluoride solution. Both treatments showed significant improvement in white spot reduction according to the microhardness enhancement in the affected teeth. However, this finding should not be interpreted as equal effectiveness. Intergroup analysis was done to determine which solution is better, and the analyzed data showed that although there are no differences between the calcium phosphate and fluoride groups, but calcium phosphate showed to be more effective in improving microhardness of white spot lesions than artificial saliva, while fluoride showed no additional benefits. Means of microhardness improvement in calcium phosphate was greater than fluoride although the difference was not significant. Several investigations have indicated the superiority of calcium phosphate in treatment of white spot lesions. Güçlü *et al.* article revealed that use of CPP-ACP paste could treat white spot lesions in permanent teeth of patients, and supplementary application of 5% sodium fluoride varnish had no beneficial effects on regression of these lesions. They believed that fluoride therapy could result in hypermineralization of surface layer in presence of fluoride ion, which blocks entering of mineralizing ion to the subsurface area (24). Kumar *et al.* revealed that use of ACP as a tooth paste or topical coating can decrease lesion depth, and combination of ACP with fluoridated toothpaste has a higher remineralization potential (25). In a systematic review, Lopatiene demonstrated that ACP and fluoride are both effective in treatment of white spot lesions during and after fixed orthodontic treatment, however CPP-ACP can be more beneficial in ameliorating these lesions (18). On the other hand, we found three studies which their results showed no difference between calcium phosphate and fluoride solution in healing of post-orthodontic white spot lesions (19,26,27). All of these three studies used quantitative light-induced fluorescence (QLF) to assess the changes between groups. QLF is an accurate device, particularly in the assessment of caries on smooth surfaces (sensitivity and specificity of 0.76 (± 0.02 [SD]) and 0.85 (± 0.09 SD) respectively). However, there are limitations in the clinical application of this device. Limited diagnostic value in determining lesions in the proximal and occlusal surface, the effect of shape, caries localization, plaque, staining and ambient light (which interfere with fluorescent light) in altering the assigned number of lesions, are potential errors in using this device. In the studies done by Andersson *et al.*, Bröchner *et al.* and Beerens *et al.*, plaque removal was mentioned but no explanation was found regarding mesioincisal and distoincisal lesions (which QLF could not determine well) and covering the ambient light. The bias of using QLF has been suggested to be removed by longer period of study with a greater sample size and identical baseline characteristics of the treatment and control group (28,29). In these three studies, the sample size seems to be adequate, but no efforts have been made to make groups as identical as possible.

In the current study, intact teeth with no defects were selected and underwent the same protocol for white spot inducement. The microhardness assessment which is an accurate method for determining mechanical properties of carious lesion was used and compared between groups. Our investigation demonstrated that physicomechanical properties of teeth treated with both fluoride and ACP improved, and although this improvement was higher in the ACP group but no statistical difference was found. Despite these results, there is more important question to respond. Whether such different in microhardness improvement can lead to higher clinical outcome or not? The answer to this question is unclear and longitudinal *in-vivo* investigations are needed. Effectiveness of fluoride in prevention of white spot lesions has been proved with strong evidence in well-designed systematic review (17). But there is no consensus which method or combination of methods of delivering fluoride is the most effective. Some investigations proposed using the combination of ACP and fluoride (27) as a probably better method for prevention and treatment of white spot lesions. Few articles showed controversy in effects of using the combination of fluoride and ACP (24,30). Orthodontists are responsible for their patients’ oral health and should prescribe these products, particularly in high risk conditions.

 Although there are investigations on the combined use of these solutions, we suggest a well-designed clinical trial to be performed to investigate the effectiveness of fluoride solution, ACP solution and combination of these two products.

## Conclusions

Fluoride solution and ACP solution enhanced the enamel microhardness of white spot lesions and it seems they are suitable products for treatment of white spot lesions.
